# Rho‐kinase pathway activation and apoptosis in circulating leucocytes in patients with heart failure with reduced ejection fraction

**DOI:** 10.1111/jcmm.14819

**Published:** 2019-11-28

**Authors:** Maria Paz Ocaranza, Jackeline Moya, Jorge E. Jalil, Sergio Lavandero, Alexis M. Kalergis, Cristián Molina, Luigi Gabrielli, Iván Godoy, Samuel Córdova, Pablo Castro, Paul Mac Nab, Victor Rossel, Lorena García, Javier González, Cristián Mancilla, Camila Fierro, Luis Farías

**Affiliations:** ^1^ Department of Cardiovascular Diseases School of Medicine Pontificia Universidad Católica de Chile Santiago Chile; ^2^ Advanced Center for Chronic Diseases (ACCDiS) School of Medicine Pontificia Universidad Católica de Chile Santiago Chile; ^3^ Faculty of Chemical and Pharmaceutical Sciences Advanced Center for Chronic Diseases (ACCDiS) Universidad de Chile Santiago Chile; ^4^ Cardiology Division Department of Internal Medicine University of Texas Southwestern Medical Center Dallas Texas; ^5^ Departament of Molecular Genetics and Microbiology Faculty of Biological Sciences Millennium Institute on Immunology and Immunotherapy Pontificia Universidad Católica de Chile Santiago Chile; ^6^ Department of Medicine Hospital del Salvador Medical School Universidad de Chile Santiago Chile

**Keywords:** apoptosis, ERM, heart failure, MYPT1, remodelling, Rho‐kinase

## Abstract

**Background:**

Increased Rho‐kinase activity in circulating leucocytes is observed in heart failure with reduced ejection fraction (HFrEF). However, there is little information in HFrEF regarding other Rho‐kinase pathway components an on the relationship between Rho‐kinase and apoptosis. Here, Rho‐kinase activation levels and phosphorylation of major downstream molecules and apoptosis levels were measured for the first time both in HFrEF patients and healthy individuals.

**Methods:**

Cross‐sectional study comparing HFrEF patients (n = 20) and healthy controls (n = 19). Rho‐kinase activity in circulating leucocytes (peripheral blood mononuclear cells, PBMCs) was determined by myosin light chain phosphatase 1 (MYPT1) and ezrin‐radixin‐moesin (ERM) phosphorylation. Rho‐kinase cascade proteins phosphorylation p38‐MAPK, myosin light chain‐2, JAK and JNK were also analysed along with apoptosis.

**Results:**

MYPT1 and ERM phosphorylation were significantly elevated in HFrEF patients, (3.9‐ and 4.8‐fold higher than in controls, respectively). JAK phosphorylation was significantly increased by 300% over controls. Phosphorylation of downstream molecules p38‐MAPK and myosin light chain‐2 was significantly higher by 360% and 490%, respectively, while JNK phosphorylation was reduced by 60%. Catecholamine and angiotensin II levels were significantly higher in HFrEF patients, while angiotensin‐(1‐9) levels were lower. Apoptosis in circulating leucocytes was significantly increased in HFrEF patients by 2.8‐fold compared with controls and significantly correlated with Rho‐kinase activation.

**Conclusion:**

Rho‐kinase pathway is activated in PMBCs from HFrEF patients despite optimal treatment, and it is closely associated with neurohormonal activation and with apoptosis. ROCK cascade inhibition might induce clinical benefits in HFrEF patients, and its assessment in PMBCs could be useful to evaluate reverse remodelling and disease regression.

## 1 INTRODUCTION

Increased neurohormonal drive in heart failure (HF), mainly as a result of sympathetic and renin‐angiotensin system (RAS) activation, significantly contributes to pathological cardiac remodelling and disease progression.^1^, ^2^ Both neurohormonal systems promote activation of the small protein RhoA signalling pathway and its target Rho‐kinase (ROCK). Activated ROCK phosphorylates and switches on several intracellular proteins, promoting cardiac hypertrophy, ventricular dysfunction, fibrosis, inflammation and apoptosis.^1^, ^2^, ^3^, ^4^, ^5^ This pathway also modulates blood pressure by regulating smooth muscle contraction.^3^, ^4^

ROCK activity in circulating leucocytes has been studied extensively in patients. Elevated ROCK activity is associated with metabolic syndrome,^6^ cigarette smoking and with endothelial dysfunction.^7^ ROCK activity in circulating leucocytes was 90% higher in patients with cardiovascular disease compared to healthy tindividuals and the ROCK inhibitor fasudil significantly increased their forearm blood flow (but not in healthy individuals).^8^ ROCK activation in peripheral blood mononuclear cells (PBMCs) has been found in human hypertension,^9^, ^10^ with higher levels in hypertensive patients with left ventricular hypertrophy (LVH).^9^ In patients with heart failure and reduced ejection fraction (HFrEF), ROCK activity in circulating leucocytes is markedly elevated^11^, ^12^, ^13^ and inversely correlated with ejection fraction.^11^

There are few data in heart failure and related conditions regarding downstream effects after ROCK activation in PBMCs. In DOCA hypertensive rats, with LVH and increased myocardial fibrosis, ROCK activation in PBMCs is significantly correlated with activation of this pathway in the myocardium and with determinants of cardiac remodelling (hypertrophy, fibrosis and inflammation).^14^ Most interestingly, ROCK activation increased levels of ROCK1 and the pro‐inflammatory molecules p65 NF‐KB, VCAM1 and IL‐6 simultaneously in the myocardium and in PBMCs that decreased to control levels with the ROCK inhibitor fasudil.^14^ Furthermore, in normotensive Brown Norway rats (BN), an preclinical model with genetically determined high angiotensin‐converting enzyme (ACE) and angiotensin II levels,^15^, ^16^ and we previously found significantly increased phosphorylation of MYPT1, ERM and p38‐MAPK as well as increased levels of p65‐NF‐κB at the same time in the LV and in PBMCs, and fasudil reduced them to levels observed in their respective controls.^17^

Observations in rodents indicate that ROCK contributes both to myocardial fibrosis and cardiomyocyte apoptosis.^18^ ROCK1 activation by caspase‐3 plays an essential role in cardiomyocyte apoptosis.^19^ In a transgenic mouse model of dilated cardiomyopathy, ROCK1 deletion attenuated left ventricular dilation, contractile dysfunction and cardiomyocyte apoptosis^20^ and cardiomyocyte‐specific ROCK1 overexpression accelerated progression to HF by increasing apoptosis and fibrosis.^21^ These studies strongly suggest that ROCK activation may promote myocardial apoptosis in human HF as well.

The aforementioned observations raise several questions concerning the pathological significance of ROCK activation in HFrEF patients, specifically about the mechanisms responsible for ROCK activation, the role of downstream ROCK molecules and the clinical consequences. Moreover, there is little information about the activity of other components of this pro‐remodelling pathway and regarding the relationship between ROCK activation and apoptosis in HFrEF patients.

Thus, in order to assess more comprehensively the pathophysiological role of the ROCK cascade activation in PBMCs in human HFrEF, the purpose of the study was to relate Rho‐kinase activation levels with the main ROCK downstream molecules associated with myocardial remodelling as well as to apoptosis levels in circulating leucocytes in patients with HFrEF. The direct relationship of ROCK activation in the myocardium and in PBMCs was further examined in a preclinical model with activation of the renin‐angiotensin system and high cardiovascular ROCK levels.^17^

## 2 METHODS

This cross‐sectional observational study compared patients with stable chronic HFrEF and healthy controls. The study adhered to the Declaration of Helsinki principles and was approved by the Human Research Ethics Committee at the Pontificia Universidad Catolica de Chile School of Medicine. Written informed consent was obtained from all participants prior to any procedure.

Participants were consecutive patients (n = 20) with stable chronic congestive HFeEF, functional class II or III (New York Heart Association), and ejection fraction ≤35% under optimal medical treatment. Stability was defined according to the following criteria: no hospitalizations or emergency room visits for HF in the last year neither deterioration in renal function, clinical tolerance to vasodilators and betablockers, no dyspnoea in daily life activities, ability to walk ≥1 block on level ground without dyspnoea or fatigue and no need to increase diuretics to keep volume status, abscence of hyponatremia. These criteria are currently used to define clinical stability in HFrEF patients.^22^ All patients were in sinus rhythm. Controls (n = 19) were healthy individuals (by medical history, symptoms, electrocardiogram and echocardiogram), matched for age and gender, not taking any antihypertensive drug. Exclusion criteria were neoplastic disease in the last 4 years, active infection in the last 8 weeks, use of high‐dose statins,^23^ any other clinically significant chronic disease, obesity and diabetes.

### 2.1 Echocardiographic measurements

Echocardiograms were obtained using a Philips iE33 instrument with a 2.5‐MHz transducer to evaluate cardiac function at the time of blood sampling. Measurements were performed by a blinded rater according to American Society of Echocardiography recommendations.^24^

### 2.2 Plasma oxidative stress

Plasma malondialdehyde (MDA) and 8‐isoprostane levels were measured in venous blood in both groups. MDA levels were measured by determining the content of thiobarbituric acid‐reactive substances.^25^ Plasma 8‐isoprostane levels were measured with an enzyme immunoassay kit (Cayman Chemical Company).

### 2.3 Rho‐kinase cascade proteins in peripheral blood mononuclear cells (Western blot)

To assess ROCK pathway activation, we determined (a) ROCK activation, by measuring phosphorylation of two direct ROCK targets: myosin light chain phosphatase 1 (MYPT1‐P/T) and ezrin‐radixin‐moesin (ERM‐P/T); (b) both ROCK1 and ROCK2 isoform levels; and (c) subsequent phosphorylation of the downstream ROCK cascade proteins p38‐MAPK, myosin light chain‐2 (MLC‐2) and c‐Jun N‐terminal kinase (JNK). Levels of the apoptosis indicator cleaved caspase‐3 and ROCK‐dependent (downstream) pro‐inflammatory molecules p65 nuclear factor κB (NFκB), vascular cell adhesion molecule 1 (VCAM‐1), intracellular adhesion molecule 1 (ICAM‐1), interleukin 6 (IL‐6) and interleukin 8 (IL‐8) were also measured. Additionally, phosphorylation of the upstream ROCK protein JAK2 (Janus kinase) was determined.

### 2.4 **Protein extraction from peripheral blood mononuclear cells**[9,11]

For isolating peripheral blood mononuclear cells (PBMCs), 5 vol of whole blood containing EDTA was poured over a 5 vol of density gradient cell separation medium (Ficoll and sodium diatrizoate, Histopaque‐1077, Sigma Chemical Co.) and centrifuged. White cells were separated, resuspended and washed in phosphate buffered saline (PBS). Upon isolation (4‐80 × 10^6^ viable cells, 95% viability), cells were resuspended in lysis buffer containing 150 mmol/L NaCl, 1% NP40, 0.5% deoxycholate, 0.1% sodium dodecyl sulphate (SDS) and 50 mmol/L Tris. Lysis buffer was supplemented with a protease inhibitor cocktail (1 μg/mL aprotinin, 1 μg/mL leupeptin and 1 mmol/L PMSF). Protein content was determined by the Lowry assay. ROCK activity and ROCK downstream target proteins were determined by Western blot.

### 2.5 Western blot analysis

Soluble protein fractions were heated 5 minutes at 95°C with SDS sample buffer (375 mmol/L Tris–HCl pH 6.8, 6% SDS, 48% glycerol, 9% 2‐mercaptoethanol and 0.03% bromophenol blue). Equal amounts of protein were loaded and separated on a 5% stacking and 8, 18% resolving SDS‐PAGE gel (80 V), and transferred into a nitrocellulose membrane at 400 A during 2 hours on ice. Blocking was performed with 5% BSA at room temperature. Blots were incubated overnight at 4°C with the primary antibody. The relative amount of protein was estimated by chemiluminescence (ECL plus kit, Perkin Elmer which contains the substrate for horseradish peroxidase, HRP). Digital images were obtained with a Syngene G‐Box automated system and analysed by densitometry using the software UN‐SCAN‐IT™ (Silk Scientific Corporation).

The blots were incubated overnight with the following primary antibodies: anti‐MYPT‐1 antibody (Cell signaling, Cat 2634); anti‐p‐MYPT‐1 antibody (Cyclex, Cat CY‐P1025); anti‐ERM antibody (Cell Signaling, Cat 3142); anti‐p‐ERM antibody (Cell Signaling, Cat 3141); anti‐p38 MAPK antibody (Cell Signaling, Cat 9212); anti‐phospho‐p38 MAPK antibody, Cell Signaling, Cat 4511); anti‐ROCK‐1 antibody (BD Bioscience, Cat 611136); anti‐ROCK‐2 antibody (BD Bioscience, Cat 610623); anti‐p65 nuclear factor κB antibody (Cell Signaling, Cat 8242); anti‐vascular cell adhesion molecule 1 antibody (Santa Cruz, Cat sc1504); anti‐intracellular adhesion molecule 1 antibody (Santa Cruz, Cat sc8439); anti‐interleukin 6 antibody (Abcam, Cat ab6672); anti‐interleukin 8 **a**ntibody (Abcam, Cat ab7747); anti‐myosin light chain 2 antibody (Cell Signaling, Cat cs3672) and anti‐phospho‐MLC‐2 antibody (Cell Signaling, Cat cs3674); anti‐JAK antibody (Cell signaling Cat cs3230); anti‐phospho JAK2 antibody (Cell signaling Cat sc3776); anti‐JNK antibody (Cell signaling cs9252); anti‐phospho JNK antibody (Cell signaling cat cs9251); and anti‐cleaved caspase‐3 antibody (Cell signaling cs9662). The blots were then washed and incubated with a secondary antibody HRP‐conjugated goat anti‐rabbit IgG (1:7500, Thermo Fisher Scientific, Cat 31466) or a goat antimouse IgG (1:10.000, Santa Cruz, Cat sc2005) for 2 hours. As a protein loading control, β‐actin (β‐actin, mouse monoclonal, 1/10000, Sigma, Cat A2228) was used. (Specific details of the different antibodies for Western blot analysis protocols are described in detail in On Line Supplement 1, Section 1).

### 2.6 Circulating catecholamines, BNP, angiotensin II, angiotensin‐(1‐9) and inflammatory cytokines IL‐6 and IL‐8

Venous blood samples were taken and immediately cooled and centrifuged at 4°C and then stored at −80°C until analysis. Laboratory evaluations included plasma B‐type natriuretic peptide (BNP), adrenaline and noradrenaline levels. BNP, Angiotensin II (Ang II) and vasodilatory peptide Ang‐(1‐9) levels were quantified using commercial ELISA kits (Sigma; Enzo Life Sciences; Cloud‐Clone Corp, respectively) according to the manufacturer instructions. Epinephrine and norepinephrine were measured by chromatography. Each sample was analysed in duplicate. The ELISA detection limits were 1.02 pg/mL for BNP (intra‐assay coefficient of variation [CV]: 7.8%‐9.9%; inter‐assay CV: 8.1%‐15%); 4.6 pg/mL for Ang II (intra‐assay CV 4.7%‐7.3%; inter‐assay CV 6.0%‐15.9%); and 2.5 pg/mL for Ang‐(1‐9) (intra‐assay CV < 10%; inter‐assay CV < 12%).

Serum levels of IL‐6 and IL‐8 were also determined using the Abcam ELISA kit according to the manufacturer's instructions.

### 2.7 Assessment of apoptosis by DNA fragmentation

PBMC (2 × 10^4^ cells) was harvested and washed once with phosphate buffered saline (PBS). The cells were then fixed in slices within an area of 1 cm^2^ with 4% paraformaldehyde (in PBS, pH 7.4, for at least 6 hours, RT) and air‐dried for 24 hours. Afterwards, cells were washed twice with PBS and incubated with permeabilization solution (0.1% Triton‐X‐100 in 0.1% sodium citrate, 15 minutes, RT). Permeabilization solution was removed, and TUNEL analysis was performed with the In Situ Cell Death Detection Kit, POD (Roche Inc). Cells with TUNEL‐positive nuclei with evidence of cromatin margination^26^, ^27^ were considered apoptotic. A total of 400 consecutive cells were counted in 20 sequential fields (40×). The total nuclei count and number of apoptotic nuclei were used to compute the percentage of apoptotic cells. Intra‐assay and inter‐assay coefficients of variation were 2.1%‐3.5% and 6.2%‐8.5%, respectively.

### 2.8 Apoptosis levels in the myocardium and PBMCs in a preclinical model of ROCK activation

Recently, we reported a significant correlation between ROCK activation in the myocardium and in circulating leucocytes in normotensive rats with genetically high vs low levels of ACE and ROCK activity (Brown Norway (BN) and Lewis rats, respectively).^17^ To better interpret the apoptosis data collected in the HF patients here, we used the same experimental model to measure at the same time apoptosis levels in PBMCs and in the left ventricle (LV) in 3 groups of male rats: Lewis, untreated BN and BN rats treated with the ROCK inhibitor fasudil, 100 mg/kg/d by gavage for 7 days (n = 7‐11/group). (Detailed experimental protocol described in described in detail in On Line Supplement 1, Section 2).

### 2.9 Types of PBMCs involved in enhanced ROCK signalling in HFrEF patients

In order to specify in which PBMCs types ROCK is becoming activated, MYPT1 phosphorylation in PBMC subpopulations was evaluated by flow cytometry. Accordingly, 500 000‐2 000 000 cells were taken per sample (n = 5 HFrEF patients and 5 controls) and kept in PBS/FBS 1%. The cells were incubated with the following antibodies: anti‐CD14 APCH7; anti‐CD19 BV421; anti‐CD4 BUV395; anti‐CD8 BB515 and anti‐CD3 Pecy7 during 30 minutes at 4°C. Then, the cells were washed with 1 mL of PBS/foetal bovine serum (FBS)1%. The samples were centrifuged at 360 *g* for 6 minutes, and the supernatant discarded and resuspended in PBS/2% PFA to be fixed for 15 minutes at 4°C. Then, the cells were washed with 1 mL of PBS/FBS 1%. The next step was to permeabilize the cells with a permeabilization buffer containing 0.1% saponin, 10% FBS, 0.1% BSA, 1 mmol/L CaCl_2_, 1 mmol/L MgSO_4_ and 40 mmol/L Hepes (50 μL to each sample). After 10 minutes of incubation in this solution, the phosphorylated and total anti‐MYPT1 antibodies were added and incubated for 45 minutes at 4°C. Afterwards, the cells were washed as mentioned above and incubated with an APC antimouse antibody that recognizes the anti‐MYPT1 antibody for one hour (4°C). After this time, the cells were washed twice with permeabilization buffer and resuspended in PBS containing 1% FBS. Finally, the samples were acquired in a LSR Fortessa X20 cytometer.

### 2.10 Statistical analysis

Data are presented as mean ± SD. Differences between mean values were compared using a *t* test, and one factor ANOVA followed by the Neuman Keuls test. Correlation analyses were performed using the Pearson correlation coefficient. *P* values ≤.05 were considered statistically significant.

## 3 RESULTS

### 3.1 Clinical characteristics, laboratory tests and cardiac remodelling assessed by echocardiography

The aetiology of HFrEF in the patient sample was mainly dilated cardiomyopathy and coronary heart disease with standard pharmacological treatment (Table 1). Demographics, heart rate, blood pressure and blood chemistry results were similar in both groups (Table 2).

**Table 1:** Functional class, aetiology and pharmacological treatment in the HFrEF patients (n = 20)

NYHA functional class	
II (%)	25
II‐III (%)	45
III (%)	30
Aetiology	
Idiopathic dilated cardiomyopathy (%)	55
Coronary heart disease (%)	25
Hypertensive (%)	15
Secondary to antraciclines (%)	5
Pharmacological treatment	
ACE inhibitors (%)	35
Angiotensin receptor blockers (%)	55
Betablockers (%)	95
Furosemide (%)	65
Spironolactone (%)	95
Digoxine (%)	15
Fibrates (%)	5
Atorvastatine (%)	40
Aspirine (%)	30

**Table 2:** Demographics, blood pressure and heart rate, blood chemistry and plasma oxidative stress levels

	HFpatients (n = 20)	Control patients (n = 19)	*P* value
Age (y)	56.9 ± 13.0	57.7 ± 5.9	>.05
Men (%)	55	63	>.05
Weight (kg)	74.5 ± 12.9	74.7 ± 8.0	>.05
Body mass index (kg/m^2^)	27.7 ± 4.0	25.9 ± 1.7	>.05
Systolic blood pressure (mm Hg)	107.0 ± 18.5	115.0 ± 11.3	>.05
Diastolic blood pressure (mm Hg)	67.4 ± 10.1	75.8 ± 5.6	.05
Heart rate (bpm)	64.3 ± 7.1	63.7 ± 9.3	>.05
Haematocrit (%)	41.9 ± 2.7	43.2 ± 3.0	>.05
Haemoglobin (mmol/L)	8.7 ± 1.0	9.1 ± 1.1	>.05
WBC/Ml	6400 ± 1057	5939 ± 1147	>.05
Absolute neutrophil count (/mm^3^)	6631 ± 1550	6200 ± 1649	>.05
Neutrophil‐to‐lymphocyte ratio	2.1 ± 1.0	2.2 ± 1.6	>.05
Plasma creatinine (μmol/L)	79.6 ± 0.2	70.7 ± 0.2	>.05
BUN (mmol/L)	7 ± 5.2	5.11 ± 3.9	≤.05
Potassium, serum (mmol/L)	4.1 ± 0.2	4.2 ± 0.3	>.05
Alanine aminotransferase (μkat/L)	0.41 ± 0.2	0.32 ± 0.1	>.05
Aspartate aminotransferase (μkat/L)	0.39 ± 0.2	0.33 ± 0.1	>.05
Uric acid (μmol/L)	333.1 ± 29.7	315.2 ± 17.8	>.05
BUN/plasma creatinine	22.3 ± 5.5	17.8 ± 3.7	≤.05
Serum cholesterol (mmol/L)	4.51 ± 1.2	5.35 ± 0.62	≤.05
LDL cholesterol (mmol/L)	2.69 ± 0.90	3.24 ± 0.75	>.05
HDL cholesterol (mmol/L)	1.05 ± 0.23	1.47 ± 0.4	≤.05
Triglicerydes (mmol/L)	1.48 ± 0.7	1.27 ± 0. 5	>.05
MDA (μM)	1.3 ± 0.9	1.1 ± 0.5	>.05
8‐Isoprostane (pg/mL)	11.1 ± 4.5.3	8.7 ± 4.3	>.05

Note

Values shown as mean ± SD.

Abbreviations: BUN, blood urea nitrogen; MDA, malondialdehyde; NS, nonsignificant; WBC, white blood cells.

Interventricular septum thickness, left atrial diameter, LV end‐diastolic diameter and pulmonary artery systolic pressure were significantly greater in the HFrEF patients, with values 13%, 29%, 40% and 61% greater than in control patients (Table 3). The LV end‐systolic diameter was 2‐fold greater in the HF patients (*P* < .01). Systolic LV function was markedly deteriorated in the HFrEF patients.

**Table 3:** Cardiac dimensions, LV systolic function and pulmonary artery systolic pressure by echocardiography

	HF patients (n = 20)	Control patients (n = 19)	*P* value
Posterior wall thickness (mm)	9.1 ± 1.8	8.7 ± 1	>.05
Septum thickness (mm)	10.1 ± 1.7	8.9 ± 1.2	≤.05
LV end‐diastolic diameter (mm)	68.4 ± 8.7	48.9 ± 5.5	≤.05
LV end‐systolic diameter (mm)	59.0 ± 9.1	29.7 ± 2.5	≤.05
Left atrial diameter (mm)	48.2 ± 7.7	37.3 ± 3.6	≤.05
LV Shortening fraction (%)	13.6 ± 6.6	37.0 ± 5.0	≤.05
LV Ejection fraction (%)	25.7 ± 6.2	67.8 ± 4.4	≤.05
Pulmonary artery systolic pressure (mm Hg)	40.9 ± 12.3	25.4 ± 1.3	≤.05

Note

Values shown as mean ± SD.

Abbreviation: LV, left ventricle.

### 3.2 Rho‐kinase activation and JAK2 phosphorylation levels (upstream of Rho‐kinase) in PBMCs (Figure 1)

**Figure 1:**
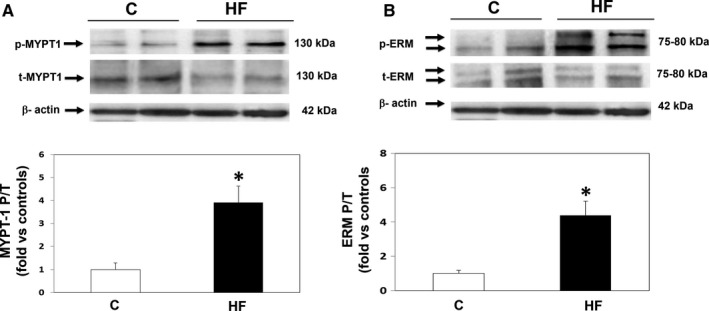
Increased ROCK activity in circulating leucocytes as measured by MYPT‐1 and ERM phosphorylation in HFrEF patients and control patients. (A) Upper panel: Representative Western blots from 2 controls (left) and 2 HFrEF patients (right). MYPT1‐P = Phosphorylated MYPT‐1, MYPT1‐T = Total MYPT‐1. Lower panel: MYPT‐1‐P/T in both groups (*P < .01 vs controls, mean ± SD; n = 15‐17 per group, statistical power = 100%). (B) Upper panel: Representative Western blots from 2 controls (left) and 2 HFrEF patients (right). ERM‐P = Phosphorylated ERM, ERM‐T = Total ERM. Lower panel: ERM‐P/T values in both groups (*P < .01 vs controls, mean ± SD; n = 19‐20 per group, statistical power = 100%)

The mean ratio between phosphorylated and total MYPT1 (MYPT1‐P/T), a measurement of ROCK activation, was significantly increased by 3.9‐fold in the HFrEF patients (*P* < .001) (Figure 1A), whereas ROCK1 and ROCK 2 isoform levels were similar in the two groups (Table 4). The MYPT1‐P/T ratio was inversely correlated with LV fractional shortening (*r *= −.66, *P* < .001), LVEF (*r *= −.53; *P* < .01) and directly correlated both with pulmonary artery systolic pressure (*r* = .55; *P* < .01) and with the end‐systolic LV diameter (*r* = .67; *P* < .01).

**Table 4:** Components of the ROCK pathway in PBMCs (Western blot) and circulating levels of cytokines IL‐6 and IL‐8, BNP, catecholamines and angiotensins

	HF patients (n = 20)	Control patients (n = 19)	*P* value
In PBMCs (blood mononuclear cells)			
ROCK 1	0.7 ± 0.4	1.0 ± 0.6	>.05
ROCK 2	1.2 ± 1.0	1.0 ± 0.5	>.05
JAK P/T	3.2 ± 1.5	1.0 ± 0.2	≤.05
IL‐6 (UOD)	5.8 ± 5.4	1.0 ± 0.7	≤.05
IL‐8 (UOD)	7.0 ± 4.5	1.0 ± 0.8	≤.05
ICAM‐1 (UOD)	2.2 ± 1.9	1.0 ± 0.6	≤.05
VCAM‐1 (UOD)	1.0 ± 0.8	1.0 ± 0.5	>.05
p65‐NFkB (UOD)	1.1 ± 0.8	1.0 ± 0.5	>.05
Circulating levels			
Brain natriuretic peptide (BNP, pg/mL)	183 ± 12.4	56 ± 11.2	≤.05
Adrenaline (pg/mL)	47 ± 27.6	25 ± 9.4	≤.05
Noradrenaline (pg/mL)	421 ± 237	243 ± 132.3	≤.05
Angiotensin II (pg/mL)	12.0 ± 3.1	9.2 ± 2.8	≤.05
Angiotensin‐(1‐9) (pg/mL)	8.5 ± 6.4	61.5 ± 34.5	≤.05
IL‐6 (pg/mL)	2.7 ± 1.9	0.4 ± 0.3	≤.05
IL‐8 (pg/mL)	3.3 ± 1.7	3.5 ± 0.5	>.05

Note

Values are shown as mean ± SD (fold vs control patients).

Abbreviations: ICAM‐1, Intercellular Adhesion Molecule 1; IL, interleukin; JAK, Janus kinase; JNK, Jun amino‐terminal kinase; MLC, myosin light chain; P/T, phosphorylated/total; p65‐NFkB, Nuclear factor NF‐kappa‐B p65 subunit; UOD, Units of optical density; VCAM‐1, Vascular Cell Adhesion Protein 1 or vascular cell adhesion molecule 1.

In addition, there was a significant 4.8‐fold increase (*P* < .001) in ERM (ezrin‐radixin‐moesin) phosphorylation, another direct ROCK substrate in HF patients (Figure 1B). ERM and MYPT1 phosphorylation levels were correlated (*r* = .47; *P* < .01).

JAK2 phosphorylation levels (upstream of Rho‐kinase) were significantly increased by 3‐fold in HFrEF patients as compared to controls (Table 4).

### 3.3 Downstream ROCK pathway components in PBMCs (Figure 2)

**Figure 2:**
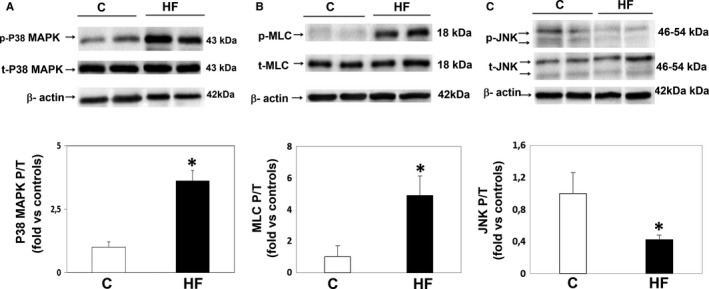
p38‐MAPK, MLC and JNK phosphorylation (Western blot) in PBMCs from HFrEF patients and control patients. (A) Upper panel: Representative Western blots from 2 controls (left) and 2 HFrEF patients (right). p38‐P = Phosphorylated p38‐MAPK, p38‐T = Total p‐38‐MAPK. Lower panel: p38‐MAPK phosphorylation in both groups (*P < .01 vs controls, mean + SD; n = 17‐19, per group, statistical power = 100%). (B) Upper panel: Representative MLC Western blots from 2 controls (left) and 2 HFrEF patients (right). Lower panel: MLC phosphorylation in both groups (*P < .01 vs controls, mean + SD; n = 17‐19, per group, statistical power = 100%). (C) Upper panel: Representative JNK Western blots from 2 controls (left) and 2 HFrEF patients (right). Lower panel: JNK phosphorylation in both groups (*P < .05 vs controls, mean + SD; n = 17‐19, per group, statistical power = 100%)

HFrEF patients showed a significant 3.6‐fold increase in p38‐MAPK phosphorylation (*P* < .001, Figure 2A) and correlated with ROCK activation assessed by MYPT1‐P/T levels (*r* = .5; *P* < .01). Moreover, in HFrEF patients MLC‐2 phosphorylation levels were significantly increased by 4.9‐fold compared with control subjects (Figure 2B), whereas JNK phosphorylation was significantly reduced by 60% in the same patients (Figure 2C).

HFrEF patients also showed a significant 2.2, 5.8 and 7‐fold increase in ICAM‐1, IL‐6 and IL‐8 levels, respectively, as compared to control subjects (Table 4), while p65‐NFkB and VCAM‐1 levels were similar in the two groups.

### 3.4 Circulating levels of BNP, catecholamines, angiotensins and inflammatory cytokines IL‐6 and IL‐8

**Figure 3:**
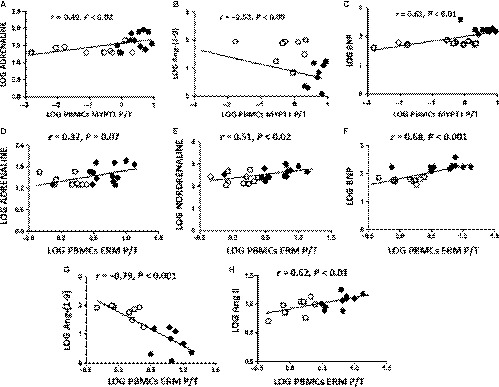
Relationship between ROCK activation measured by PBMCs MYPT‐1 and ERM phosphorylation and circulating levels of cathecolamines, BNP, Ang‐(1‐9), Ang II and in patients with HFrEF (*black circles*) and in control patients (*white circles*). Linear relationship assessed with the Spearman correlation coefficient. (A) Relationship between log PBMC MYPT‐1 phosphorylation levels and log adrenaline circulating levels (*r* = .57, *P* < .01; n = 12‐15 per group). (B). Relationship between log PBMC MYPT‐1 phosphorylation levels and log Ang‐(1‐9) circulating levels (*r* = −.52, *P* < .03; n = 12‐15 per group). (C). Relationship between log PBMC MYPT‐1 phosphorylation levels and log BNP circulating levels (*r* = .61, *P* < .01; n = 12‐15, per group). (D). Relationship between log PBMC ERM phosphorylation levels and log adrenaline circulating levels (*r* = .43, *P* < .05; n = 12‐15, per group). (E). Relationship between log PBMC ERM phosphorylation levels and log noradrenaline circulating levels (*r* = .49, *P* < .05; n = 12‐15, per group). (F). Relationship between log PBMC ERM phosphorylation levels and log BNP circulating levels (*r* = .71, *P* < .001; n = 12‐15, per group). (G). Relationship between log PBMC ERM phosphorylation levels and log Ang‐(1‐9) circulating levels (*r* = −.83, *P* < .01; n = 12‐15, per group). (H). Relationship between log PBMC ERM phosphorylation levels and log Ang II circulating levels (*r* = .60, *P* < .01; n = 12‐15, per group)

Although the HFrEF patients were clinically stable and receiving complete pharmacological treatment, their BNP, adrenaline, noradrenaline and Ang II plasma levels were significantly increased compared with those in controls by 227%, 88%, 73% and 30%, respectively (Table 4). Conversely, circulating levels of the vasodilatory peptide Ang‐(1‐9) were significantly lower by 86% in the HFrEF patients compared with control subjects (Table 4). Levels of ROCK activation in PBMCs were directly correlated with most of circulating cathecolamines, Ang II and BNP levels and inversely correlated with angiotensin‐(1‐9) levels (Figure 3).

Compared with healthy controls, HFrEF patients had a 6‐fold significant increase in serum IL‐6 levels and similar serum IL‐8 levels (Table 4). Serum IL‐6 levels were correlated with MYPT1 P/T and with ERM P/T in PBMCs (*r* = .45; *P* = .06 and *r* = .61; *P* < .01, respectively).

### 3.5 Apoptosis in HFrEF patients in circulating leucocytes (Figures 4 and 5)

**Figure 4:**
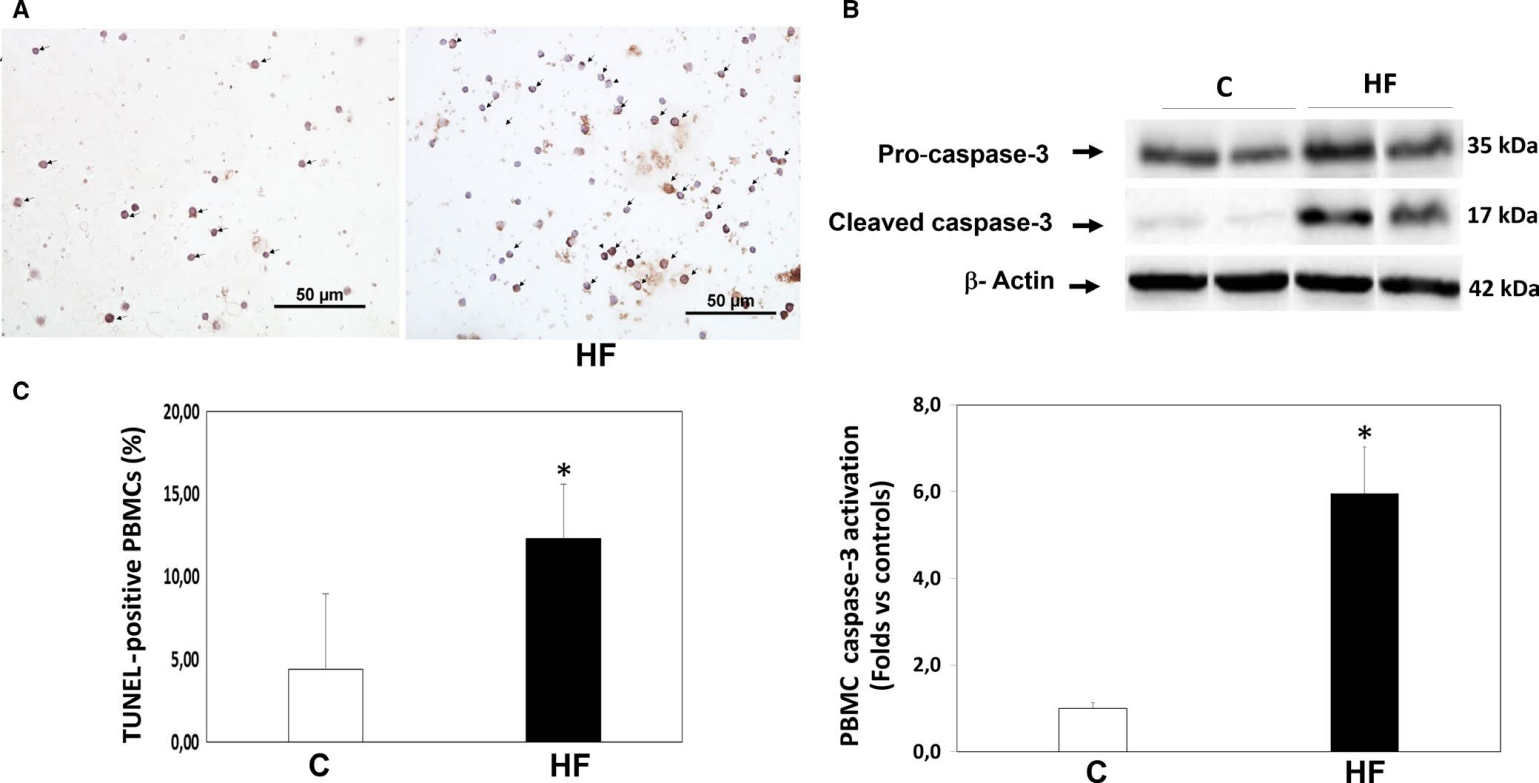
Apoptosis levels in circulating leucocytes in HFrEF patients (HF) and in control (C) patients (TUNEL analysis and cleaved caspase‐3 levels). (A) Upper panel: Representative TUNEL‐positive nuclei in one control subject (left) and one HFrEF patient (right). Bar = 50 μm Lower panel: TUNEL‐positive nuclei in controls and HFrEF patients (*P < .01 vs controls, mean + SD; n = 12‐15 per group, statistical power = 100%). (B) Upper panel: Representative bands of pro‐ and cleaved caspase‐3 subjected to the Western blot assay from 2 controls (left) and 2 HFrEF patients (right). Lower panel: Caspase‐3 activation is shown as the ratio of caspase‐3 to pro‐caspase‐3. *P < .01 vs controls, mean ± SD; n = 12‐15, per group, statistical power = 100%)

**Figure 5:**
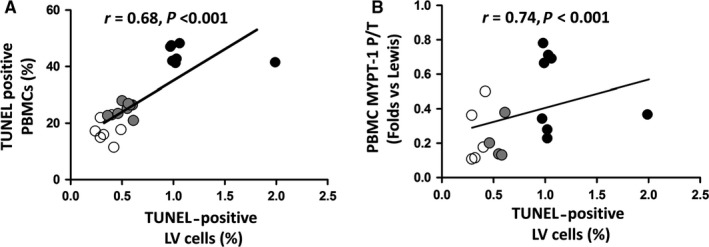
Simultaneous apoptosis levels and ROCK activation in PBMCs in patients with HFrEF and in control patients. Linear relationship assessed with the Spearman correlation coefficient. (A) Relationship between PBMCs apoptotic nuclei (%) and PBMCs MYPT‐1 phosphorylation levels (*r* = .62; *P* < .01; n = 12‐15, per group); (B) Relationship between PBMCs cleaved caspase‐3 levels and PBMCs MYPT‐1 phosphorylation levels (*r* = .57; *P* < .01; n = 12‐15, per group). Symbols: white circles = Control patients; black circles = HFrEF patients

Patients with HFrEF displayed a significant increase in apoptosis levels determined by TUNEL in their PBMCs compared to control patients (12.3 ± 3.3% vs 4.4 ± 4.6% apoptotic nuclei, *P* < .001, Figure 4A). Apoptosis assessed by TUNEL was consistent with a 5.9‐fold increase in cleaved caspase‐3 protein levels measured simultaneously in their PMBCs (*P* < .01, Figure 4B). Apoptosis levels in PBMCs assessed by TUNEL assay and by cleaved caspase‐3 measurements were significantly correlated with ROCK activation levels measured both by MYPT1 and by ERM phosphorylation levels (Figure 5).

### 3.6 Apoptosis in the myocardium and in PBMCs in a preclinical model of ROCK activation (Figures 6 and 7)

**Figure 6:**
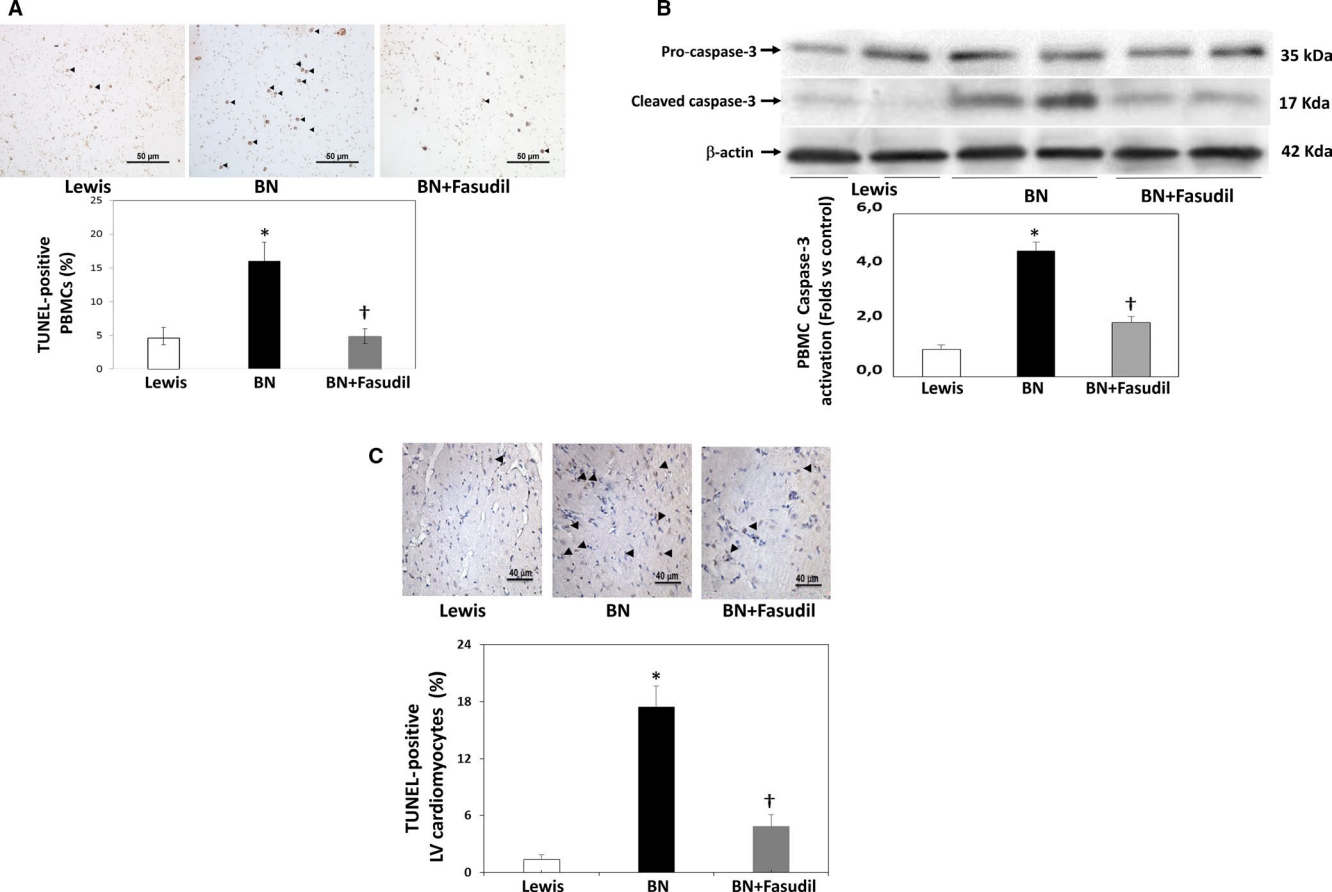
ROCK‐dependent increased apoptosis in circulating leucocytes and in the myocardium in rats with genetically determined high angiotensin‐converting enzyme and ROCK activation levels (Brown Norway, BN), BN treated with fasudil and in Lewis rats (genetically determined low angiotensin‐converting enzyme and ROCK activation levels). (A) TUNEL‐positive nuclei in PBMCs. Upper panel: Representative TUNEL‐positive nuclei in one Lewis rat (low ACE levels, low ROCK activity), one untreated BN rat (high ACE levels, high ROCK activity), and one BN rat treated with fasudil (100 mg/kg/d) for 7 d. Bar = 50 μm Lower panel: TUNEL‐positive nuclei in Lewis, untreated BN and BN rats treated with fasudil. (B) Cleaved caspase‐3 protein expression in circulating leucocytes. Upper panel: Representative bands of pro‐ and cleaved caspase‐3 (Western blots) from 2 Lewis, 2 untreated BN, and 2 BN rats treated with the ROCK inhibitor fasudil (100 mg/kg/d) for 7 d. Lower panel: Caspase‐3 activation shown as the ratio of caspase‐3 to pro‐caspase‐3 in Lewis, untreated BN and BN rats treated with fasudil. (C) TUNEL‐positive nuclei in the left myocardium. Upper panel: Representative TUNEL‐positive nuclei in left myocardium of one Lewis, one untreated BN and one BN rat treated with the ROCK inhibitor fasudil (100 mg/kg/d) for 7 d. Bar = 40 μm Lower panel: TUNEL‐positive nuclei (%) in Lewis, untreated BN and BN rats treated with fasudil. Data are shown mean + SD. Symbols: *P < .01 vs controls; †*P* < .01 vs BN after significant ANOVA (n = 7‐11/group)

**Figure 7:**
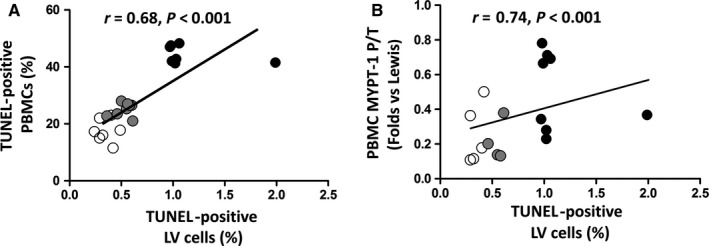
Simultaneous apoptosis levels in PBMCs and in the myocardium in rats with genetically determined high angiotensin‐converting enzyme and ROCK activation levels (Brown Norway, BN, *black circles*), BN treated with fasudil for 7 d (*grey circles*) and Lewis rats (genetically determined low angiotensin‐converting enzyme and ROCK activation levels, *white circles*). Linear relationship assessed with the Spearman correlation coefficient. (A) Relationship between PBMCs apoptotic nuclei (%) and LV apoptotic nuclei (%), (n = 7‐11 per group); (B) Correlation between LV apoptotic nuclei (%) and PBMC MYPT‐1 phosphorylation levels; *P* < .001; n = 7‐11, per group)

In the preclinical model of ROCK activation in the BN rats (with genetically determined high ACE levels and ROCK activation),^15^ significantly increased apoptosis levels both in PBMCs (by Tunel and cleaved caspase‐3 levels) (Figure 6A,B) and in the LV myocardium (Figure 6C) were observed compared to the Lewis rats (with genetically determined low levels of ACE and ROCK activation). By administering the ROCK inhibitor fasudil for 7 days to BN rats, apoptosis was significantly reduced to control (Lewis rats) levels suggesting strongly a causal relationship with ROCK activation (Figure 6). In this model, a significant correlation was found between apoptotic nuclei in PBMCs and in the LV myocardium (Figure 7A). Additionally, a significant correlation was observed between LV apoptotic nuclei with MYPT1 phosphorylation levels in PBMCs (Figure 7B).

### 3.7 Types of PBMCs involved in enhanced ROCK signalling in HFrEF patients (Figures 8 and 9)

**Figure 8:**
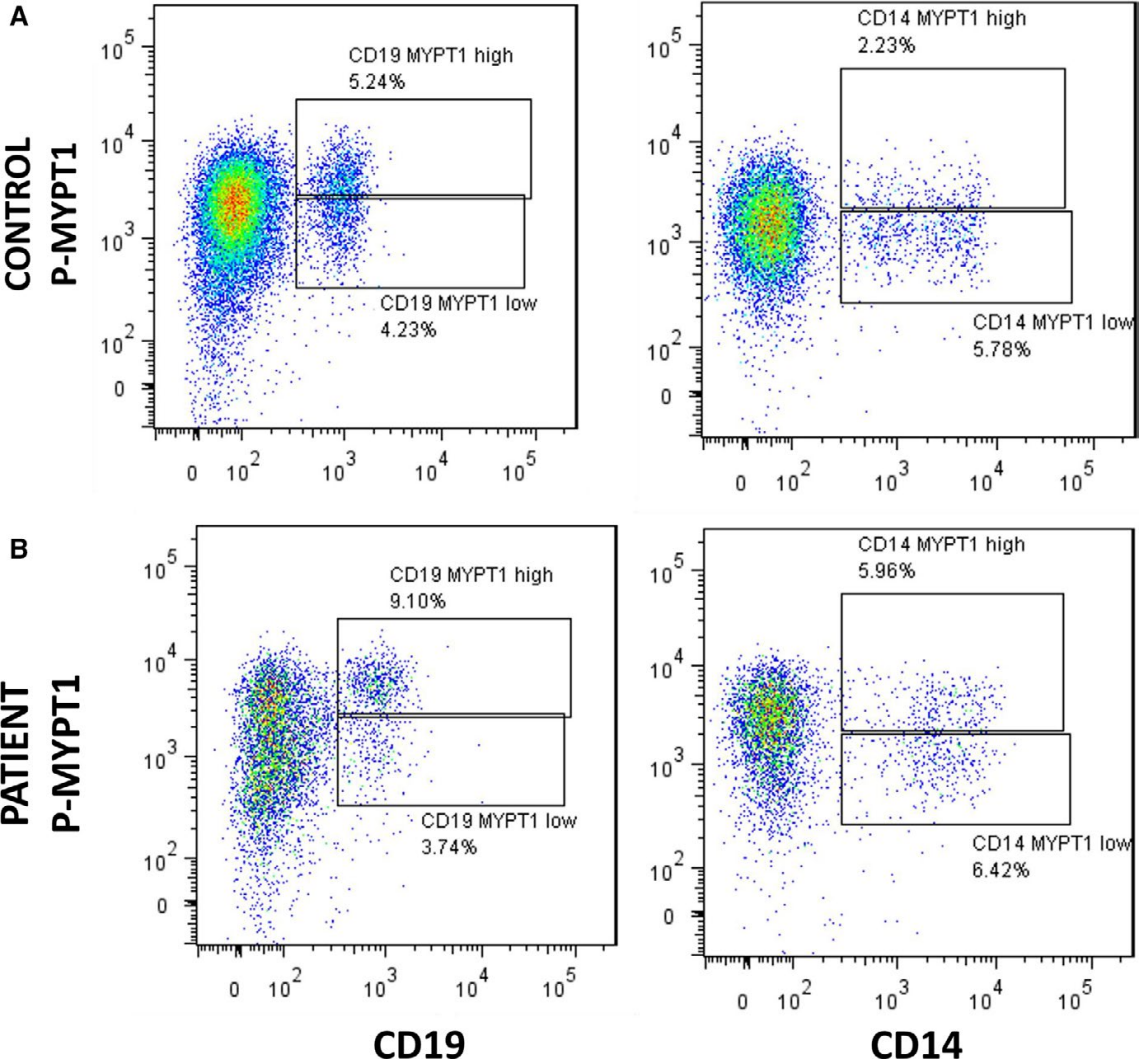
Flow cytometry of CD14 and CD19 positive cells from one HFrEF patient and a control patient. The figure shows a representative flow cytometry from a patient with HFrEF and a control patient. Expression of phosphorylated MYPT1 was measured in CD14 monocytes and CD19 B lymphocytes. Two positive populations were selected for MYPT1 phosphorylation levels, R1 and R2 (CD19 B lymphocytes) and R3 and R4 (CD14 monocytes). R1 and R3 populations have higher expression of phosphorylated MYPT1, whereas the R2 and R4 populations are those with lower expression of phosphorylated MYPT1

**Figure 9:**
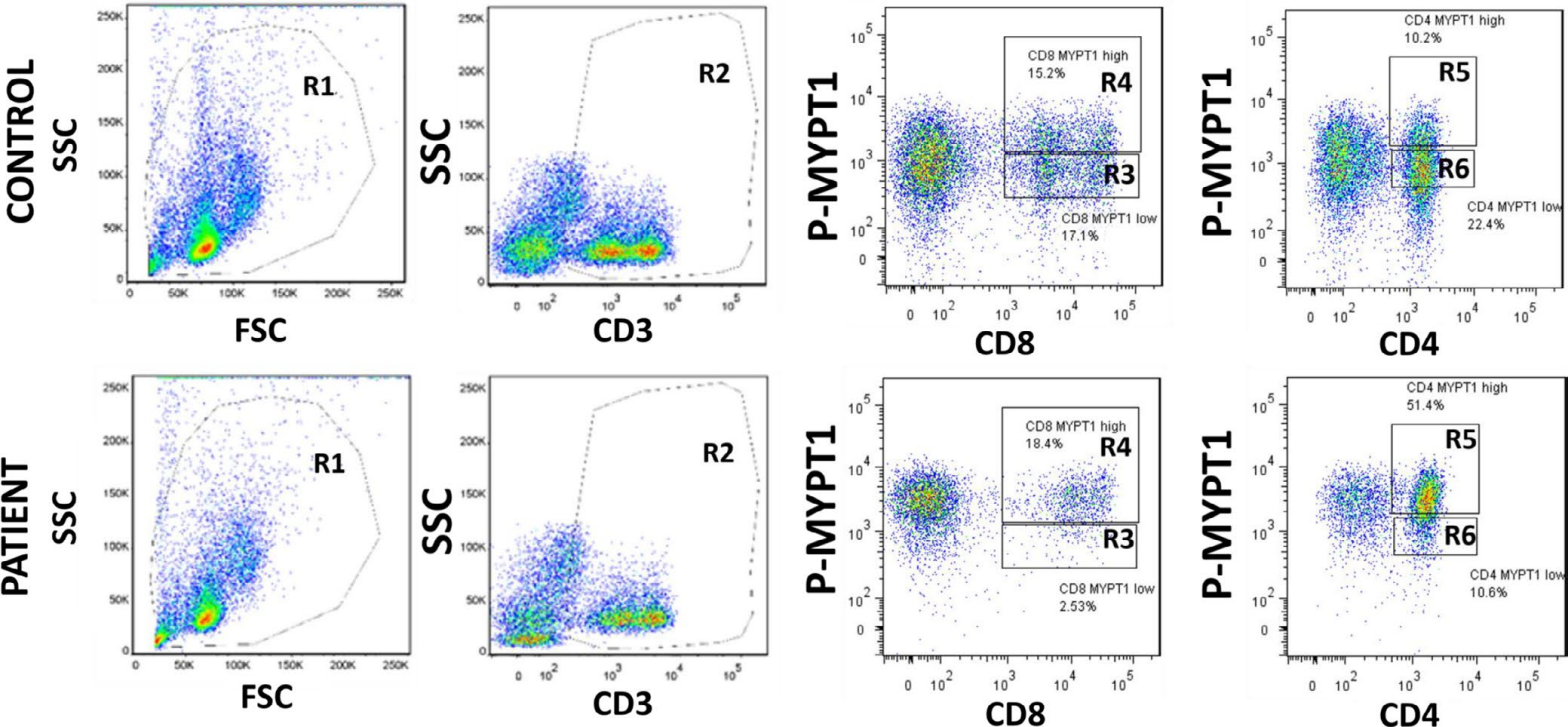
Flow cytometry of CD3 positive cells from one patient with HFrEF and a control subject. The figure shows a representative flow cytometry from a patient with HFrEF and a control patient, where the expression of phosphorylated MYPT1 was measured. The first selection was the R1 region, which represents the blood cells according to size (FSC) and cellular complexity (SSC). Then, the selection of CD3 positive cells (R2) was made, which corresponds to the population of total T lymphocytes. Subsequently, two positive populations were selected for MYPT1 phosphorylation, R3 and R4 in CD8 T lymphocytes and R5 and R6 in CD4 T lymphocytes. R3 and R5 populations are those with higher expression of phosphorylated MYPT1, whereas populations R4 and R6 are those with lower expression MYPT1 phosphorylation. As shown in the figure, the patient's blood has a higher expression of high phosphorylated MYPT1 compared with the control patient, which means that in these two cell populations, both CD4 and CD8 T lymphocytes have a higher expression of MYPT1 phosphorylated

Flow cytometry analyses in 5 consecutive HFrEF patients and controls showed that B lymphocytes (CD19+, Figure 8A,B) and monocytes (CD14+, Figure 8A,B) have low levels of MYPT1 phosphorylation compared with T lymphocytes (CD3 positive cells, Figure 9). Two positive populations were selected for phosphorylated MYPT1, R3 and R4 in the case of CD8 T lymphocytes and R5 and R6 in the case of CD4 T lymphocytes. R3 and R5 populations displayed higher expression levels of phosphorylated MYPT1, whereas R4 and R6 populations displayed lower levels of MYPT1 phosphorylation. As shown in Figure 9, the patient's PBMCs have higher levels of MYPT1 phosphorylation compared with the control samples, which means that these two cell populations, CD4 and CD8 T lymphocytes, have a higher expression of MYPT1 phosphorylated (Figure 9).

## 4 DISCUSSION

This study examined levels of key RhoA/ROCK pro‐remodelling signalling pathway molecules in PMBCs from HFrEF patients. Significant novel findings include increased phosphorylation levels of the direct ROCK target ERM, the upstream ROCK protein JAK2, and the downstream ROCK pathway molecules p38‐MAPK and MLC‐2 in HFrEF patients. These abnormalities in the ROCK cascade in PMBCs were associated with increased neurohormonal activation and most interestingly with cell death induced by apoptosis.

In searching for a surrogate tissue, tracking the ROCK cardiac remodelling pathway we turned to PBMCs, as integral to molecular and cellular mechanisms of tissue repair and cardiac pathology. Rationale for targeting PBMCs as a putative surrogate for myocardial remodelling is based on several issues: (a) The gene expression profile of multiple tissues is shared with PBMCs^28^; (b) PBMCs are central to the neuroendocrine‐immune system interface involved during stressor states; (c) PBMCs activation reveals molecular signatures in chronic HF and predicts its severity^29^, ^30^, ^31^, ^32^; (d) previously we identified the activation of PBMC ROCK proteomic in rats^14^, ^17^; and (e) the availability of PBMCs for clinical interrogation as a noninvasive target/marker able to be monitored serially.

### 4.1 ROCK activation in PMBCs, cardiac remodelling and neurohormonal status in HF

Higher ROCK activation is observed in acute compared to stable HF and in patients with HF and preserved systolic function^13^ and is related to mortality.^12^, ^13^ Besides, similar high ROCK activation levels are found in HF patients with different etiologies.^13^ In our HFrEF patients, ROCK activation was increased by 4‐fold compared to controls. Although clinically stable under treatment, the patients had elevated levels of cathecolamines and Ang II, suggesting a link between ROCK activation and neurohormonal activation. A lack of systemic oxidative stress in these patients with HFrEF was in keeping with their clinical stability.

Because several factors influence ROCK activation, the clinical significance of the ROCK signalling in PMBCs is likely connected to the integration of multiple factors causing HF. According to Watanabe et al,^33^ because biomarkers reflecting extracardiac pathologies in HF have not been defined, Rho‐kinase activity in PMBCs might emerge as a preferred biomarker for the integrated features of HF. They also point out that according to preclinical evidence, Rho‐kinase activation itself appears to contribute to the pathogenesis of HF, specifically to cardiac remodelling induced by ischaemia or hypertrophic stress leading to cardiac decompensation and HF.^33^

Cathecolamines and Ang II activate RhoA via the membrane G protein‐coupled receptor, and ROCK is an effector of active RhoA.^1^ Ang II infusion in rats causes cardiac hypertrophy, which is suppressed by fasudil.^34^ In DOCA hypertensive rats, fasudil reduces blood pressure and angiotensin II and increases Ang‐(1‐9) levels.^35^

ROCK is activated by numerous factors,^1^, ^2^, ^3^, ^4^, ^5^ and its clinical significance may involve several pathways that lead to the onset and progression of HF by promoting myocardial remodelling. ROCK activity in PMBCs increases progressively in hypertensive patients as LVH develops.^9^ In the current HFrEF patients, ROCK pathway activation in PMBCs was related to adverse cardiac remodelling, elevated levels of cathecolamines and Ang II as well as to reduced levels of Ang‐(1‐9), a novel endogenous vasodilatory and cardioprotective angiotensin.^36^, ^37^, ^38^ Thus, ROCK activity in PMBCs remained high despite optimal treatment as long as neurohormonal activation was not suppressed and myocardial remodelling not reversed.

### 4.2 ROCK pathway activation in HFrEF patients

Circulating PMBCs from HFrEF patients displayed increased phosphorylation levels of the upstream ROCK cascade molecule JAK2, of two direct ROCK targets (MYPT‐1 and ERM), the downstream molecules p38‐MAPK and MLC‐2, and higher levels of the pro‐inflammatory molecules ICAM‐1, Il‐6 and IL‐8. Reduced JNK phosphorylation levels were also found in the same cells (Table 4). Previous clinical studies in HF patients have not assessed the ROCK cascade in PBMCs but only myosin phosphatase phosphorylation.^11^, ^12^, ^13^

Our findings here also show for the first time that in patients with HFrEF, ROCK activation in PBMCs takes place predominantly in T lymphocytes, specifically in CD4 and in CD8 T lymphocytes, which is possibly related to the higher levels of ROCK‐dependent pro‐inflammatory molecules ICAM‐1, Il‐6 and IL‐8. In patients with systemic inflammatory diseases, increased ROCK activity is also observed in circulating T lymphocytes.^39^

ERM phosphorylation in humans has not been assessed previously, and its pathogenic role in HF remains unknown. In rodents with LV, systolic dysfunction increased phosphorylated ERM levels are observed in the myocardium and are normalized with fasudil, in parallel with reverse cardiac remodeling.^40^, ^41^ A significant ROCK‐dependent correlation between ERM phosphorylation in the myocardium and in PBMCs was observed previously in normotensive rats with genetically increased ACE and Ang II levels,^17^ which is consistent with the concept of myocardial ROCK activation mirrored in circulating leucocytes.

Mitogen‐activated protein kinases (MAPKs) participate in the development of cardiac hypertrophy, remodelling, contractile dysfunction and HF.^42^ In mice, increased cardiac p38‐MAPK expression is associated with reduced contractility and cardiomyopathy.^43^ In rats with cardiac remodelling induced by endurance exercise, fasudil reduces cardiac hypertrophy, apoptosis, myocardial fibrosis and myocardial p38‐MAPK levels.^44^ Besides, a significant correlation between leucocyte p38‐MAPK phosphorylation levels and cardiac and aortic wall p38‐MAPK phosphorylation levels secondary to ROCK activation was found in rats.^17^ Until now, increased p38‐MAPK phosphorylation in leucocytes in parallel with ROCK activation has not been reported in HFrEF patients.

MLC‐2 phosphorylation in circulating leucocytes was increased in the HFrEF patients. MLC‐2 is a ROCK target related to cell contraction. In human atria muscle, alpha1 adrenergic receptors mediate a relevant increase in contractile force that depends on MLCK activity, which is accompanied by an increase in MLC‐2 phosphorylation.^45^

### 4.3 Apoptosis and ROCK activation in circulating leucocytes in HFrEF

Apoptosis is a major mechanism of HF, and increased levels of TUNEL‐positive nuclei and cleaved caspase‐3 in the circulating leucocytes of HFrEF patients compared with controls were found here. Additionally, ROCK induces apoptosis in several tissues and cells.^46^, ^47^, ^48^

ROCK inhibition reduces apoptosis induced by doxorubicin of human cardiac stem cells possibly involving the cleaved caspase‐3 and ROCK/actin,^49^ and it attenuates left ventricular remodelling due to chronic intermittent hypoxia in rats by suppressing myocardial apoptosis and inflammation.^50^ Adittionally, the ROCK1/p53/NOXA signalling pathway mediates cardiomyocyte apoptosis in response to high glucose.^51^

There are no data regarding apoptosis in circulating leucocytes in human H,F and its clinical significance is challenging. Here, ROCK activation in the HFrEF patients was positively correlated with apoptosis in PMBCs.

In order to better understand the significance of increased apoptosis levels in PMBCs in our HF patients, we employed an experimental model of simultaneous cardiac and PMBCs ROCK activation.^17^ ROCK‐dependent increased apoptosis levels both in the myocardium and in PBMCs were observed (because fasudil normalized apoptosis levels in BN rats, Figure 6). In this model, apoptosis in PBMCs and in the myocardium was correlated with ROCK activation in PBMCs (Figure 7). Even though this observation is in a different pathophysiological context, the results allow us to raise the hypothesis that apoptosis in leucocytes of HF patients is consistent with myocardial apoptosis.

Various degrees of LVH have been described in patients with dilated cardiomyopathy, and LVH in them seems to have a better prognosis than in patients without LVH.^52^, ^53^, ^54^ Clinical studies have identified a number of aetiologic factors that contribute to dilated cardiomyopathy, including viral infection, alcohol consumption and microcirculatory failure, and the variation in the extent of LVH may reflect different etiologies or different stages of a progressive disease.^55^ Patients with dilated cardiomyopathy with LVH show increased LV fractional shortening response to isoproterenol than those without LVH, indicating that cardiac systolic reserve is maintained in response to beta‐adrenergic stimulation and that the beta‐adrenergic receptor pathway is more severely impaired in dilated cardiomyopathy without LVH.^55^ In our current study, mild but significantly increased posterior wall thickness observed in the patients with HFrEF with predominantly dilated cardiomyopathy is most probably the result of remodelling because of cardiomyocyte hypertrophy, increased myocardial fibrosis and inflammation associated with ROCK activation.

### 4.4 Study limitations

This was an observational study, without interventions performed in the HFrEF patients and a relatively small n size. However, statistical power for detecting the observed differences in the ROCK cascade and apoptosis in PBMCs was high. Another theoretical limitation in this study included a lack of analysis of the functional impact of circulating neutrophils on ROCK activity. To isolate blood cells, we used ficoll gradient, which selects PBMCs including both lymphocytes (T and B) and monocytes but not neutrophils. Neutrophils can influence T cell activation^56^ and produce a marked loss of CD3+ and CD4+ T cells.^57^ Thus, to determine the influence of neutrophils on ROCK activity, it is necessary to isolate lymphocytes/monocytes from neutrophils. On the other side, HF patients in the current study were clinically stable and in similar patients we previously compared ROCK activation with healthy controls and also with a second control group of hypertensive patients under pharmacological treatment and observed similar ROCK activation levels as compared to healthy controls, both control groups significantly lower compared to the HFrEF group,^11^ which is consistent with subsequent findings reported by Dong et al.^12^

From a clinical point of view, the current findings strongly suggest that ROCK activation determined in PMBCs may be useful to assess reverse remodelling and disease regression in this severe and highly prevalent clinical condition and could have prognostic relevance for forecasting outcome and monitoring effective treatment. Currently, there are no ROCK inhibitors approved for clinical use in HFrEF. However, the findings provide a better understanding of the complex adverse remodelling process in the HF population and raise the possibility of therapeutic targets involving ROCK inhibition to promote reverse remodelling and clinical benefit in patients with HFrEF.

## CONFLICT OF INTEREST

None.

## AUTHORS CONTRIBUTION

Maria Paz Ocaranza, Jackeline Moya, Jorge E. Jalil, Sergio Lavandero, Luigi Gabrielli, Iván Godoy, Samuel Córdova, Pablo F. Castro, Paul Mac Nab, Victor Rossel, Lorena García, Javier González and Camila Fierro contributed to data analysis. Maria Paz Ocaranza contributed to study coordination. Maria Paz Ocaranza contributed to manuscript writing. Jackeline Moya, Cristián Molina and Camila Fierro contributed to biochemical and molecular work. Jackeline Moya, Cristián Molina and Cristián Mancilla contributed to data analysis and discussion. Jorge E. Jalil contributed to study design and conduction. Jorge E. Jalil and Sergio Lavandero contributed to final manuscript writing. Alexis M. Kalergis, Luigi Gabrielli, Iván Godoy, Samuel Córdova, Paul Mac Nab, Victor Rossel, Lorena García, Camila Fierro and Luis Farías contributed to manuscript discussion. Alexis M. Kalergis contributed to flow cytometry data analysis. Luigi Gabrielli, Iván Godoy, Samuel Córdova and Paul Mac Nab contributed to echocardiographic work. Pablo F. Castro and Victor Rossel contributed to clinical work. Cristián Mancilla contributed to animal experiments. Cristián Mancilla contributed to samples management. Luis Farías contributed to data management and analysis.

## Data Availability

The data that support the findings of this study are available from the corresponding author upon request.
