# Supplementary material for: Rho‐kinase pathway activation and apoptosis in circulating leucocytes in patients with heart failure with reduced ejection fraction
Source: J Cell Mol Med. 2019 Nov 28;24(2):1413–27. doi: 10.1111/jcmm.14819 (PMC6991691; doi:10.1111/jcmm.14819)
Supplement: Supplementary file 1 [file JCMM-24-1413-s001.docx]

***Supplementary Data File***

**SUPPLEMENTARY METHODS**

**SECTION 1**

**Antibodies used to determine Rho-kinase activity and ROCK pathway proteins by Western Blot in human leukocytes**^1,2^**.**

The blots were incubated overnight with the following primary antibodies: anti-MYPT-1 antibody (rabbit polyclonal, 1/500 cell signaling, Cat 2634); anti-p-MYPT-1 antibody (phospho-MYPT1-Thr 853 rabbit polyclonal, 1/500, cyclex, Cat CY-P1025), anti-ERM antibody (total-ERM, rabbit polyclonal, 1/700, Cell Signaling, Cat 3142); anti-p-ERM antibody (phospho-ERM, ezrin Thr567, radixin Thr564, moesin Thr558, rabbit polyclonal, 1/700, Cell Signaling, Cat 3141); anti-p38 MAPK antibody (p-38, rabbit polyclonal, 1/1000, Cell Signaling, Cat 9212); anti-phospho-p38 MAPK antibody (Thr180/Tyr182) (phospho-p-38, rabbit monoclonal, 1/1000, Cell Signaling, Cat 4511); anti-ROCK-1 antibody (mouse monoclonal, 1/500, BD Bioscience, Cat 611136); anti-ROCK-2 antibody (mouse monoclonal, 1/2000, BD Bioscience, Cat 610623); anti-p65 nuclear factor κB antibody (NFκB, rabbit polyclonal, 1/1000, Cell Signaling, Cat 8242); anti-vascular cell adhesion molecule 1 antibody (VCAM-1, goat polyclonal, 1/1000, Santa Cruz, Cat sc1504); anti-intracellular adhesion molecule 1 antibody (ICAM-1, mouse monoclonal, 1/1000, Santa Cruz, Cat sc8439); anti-interleukin 6 antibody (IL-6, rabbit polyclonal, 1/500, Abcam, Cat ab6672); anti-interleukin 8 **a**ntibody (IL-8, rabbit polyclonal, 1/500, Abcam, Cat ab7747); anti-myosin light chain 2 antibody (MLC-2, rabbit polyclonal, 1/1000, Cell Signaling, Cat cs3672) and anti-phospho-MLC-2 antibody (Thr18/Ser19) (p-MLC-2, rabbit polyclonal, 1/500, Cell Signaling, Cat cs3674); anti JAK antibody (JAK, rabbit polyclonal, 1:1000 Cell signaling Cat cs3230); anti-phospho JAK2 antibody (p-JAK, 1:500 Rabbit polyclonal Cell signaling Cat sc3776); anti JNK antibody (JNK, rabbit polyclonal, 1:1000 cell signaling cs9252); anti-phospho JNK antibody (p-JNK, 1:500 rabbit polyclonal Cell signaling cat cs9251); anti cleaved caspase 3 antibody (caspase 3, rabbit polyclonal, 1:1000 Cell signaling cs9662). The blots were then washed and incubated with a secondary antibody HRP-conjugated goat anti-rabbit IgG (1:7500, Thermo Fisher Scientific, Cat 31466) or a goat anti-mouse IgG (1:10.000, Santa Cruz, Cat sc2005) for 2 h. As a protein loading control, β-actin (β-actin, mouse monoclonal, 1/10000, Sigma, Cat A2228) was used.

**SECTION 2**

**Experimental Protocol to determine Apoptosis levels in the myocardium and in circulating leukocytes in a preclinical ROCK activation model**

Experiments were conducted following the “Guide for the Care and Use of Laboratory Animals” (NIH No. 85-23, revised 1996) and were approved by the Ethics Committee on Animal Welfare of the Faculty of Medicine, Pontificia Universidad Católica de Chile.

**2a. Genetically determined high versus low angiotensin converting enzyme model (and high vs low ROCK activation levels, respectively).** Homozygous adult male rats (age: 12 weeks) with genetically low ACE levels (Lewis rats) and genetically high ACE levels (Brown Norway, BN)^3,4^, were obtained from our animal facility. The homozygous condition from the BN and Lewis groups was confirmed by PCR as described^3,4^. Rats were kept under common light/dark cycles with regulated temperature and humidity, and free access to water and food. One group of BN rats received oral fasudil (BN + Fasudil; 100 mg kg^−1^ day^−1^ by gavage for 7days). The other BN group received just vehicle by gavage during 7 days. One week after administering fasudil or vehicle the animals were euthanized by deep anesthesia (ketamine HCl/xylazine 35/7 mg kg−1 i.p.).

### 2b. Evaluation of apoptotic circulating leucocytes by DNA fragmentation in circulating leukocytes

Immediately after euthanasia, blood was collected by cardiac puncture and leucocytes (2x10^4^ cells) were harvested and washed once with PBS. The cells were then fixed in slices within an area of 1 cm^2^ with 4% paraformaldehyde (in PBS, pH 7.4, at least 6 h, RT) and were air-dried on the slides for 24 h. Afterwards, the air-dried cells were washed twice with PBS, and incubated in permeabilization solution (0.1% Triton-X-100 in 0.1% sodium citrate, 15 min, RT). The permeabilization solution was then removed and the terminal deoxynucleotidyltransferase-mediated dUTP nick end labeling (TUNEL) analysis was performed with the in situ cell death detection kit POD (Roche In, Indianapolis, USA) according to manufacturer’s instructions. Background was assessed in comparison to both the negative control (leucocytes exposed to the reaction mixture without terminal deoxynucleotidyl transferase) and the positive control (leucocytes pretreated with DNase I). Cells with TUNEL-positive nuclei were considered apoptotic. A minimum of twenty fields per slide was used to calculate the percentage of apoptotic cells. 400 consecutive cells were counted in 20 sequential fields (40x). The total nuclei count and the number of apoptotic nuclei were used to compute the percentage of apoptotic cells.

**2.c Determination of cleaved caspase-3 levels in circulating leukocytes**

**Protein extraction from circulating leukocytes and Western blot analysis**

Without delay, after euthanasia blood was also collected by cardiac puncture in EDTA-containing tubes. For isolating circulating leukocytes, 5 vol of whole blood containing EDTA was poured over a 5 vol of density gradient cell separation medium (Ficoll and sodium diatrizoate, Histopaque-1077, Sigma Chemical Co., St Louis, MO) and centrifuged. White cells were separated, resuspended and washed in phosphate buffered saline (PBS). Upon isolation (4-80×10^6^ viable cells, 95% viability), cells were resuspended in lysis buffer containing 150 mM NaCl, 1% NP40, 0.5% deoxycholate, 0.1% sodium dodecyl sulfate (SDS) and 50 mM Tris. Lysis buffer was supplemented with a protease inhibitor cocktail (1 μg/ml aprotinin, 1 μg/ml leupeptin and 1 mM PMSF). Protein content was determined by the Lowry assay.

Soluble protein fraction was heated 5 min at 95 °C with SDS sample buffer (375mM Tris–HCl pH 6.8, 6% SDS, 48% glycerol, 9% 2-mercaptoethanol and 0.03% bromophenol blue). Equal amounts of protein were loaded and separated on a 5% stacking and 8, 18% resolving SDS-PAGE gel (80V), and transferred into a nitrocellulose membrane at 400 A during 2 h on ice. Blocking was performed with 5% BSA at room temperature. Blots were incubated overnight at 4 °C with the primary antibody. Relative amount of protein was determined by chemiluminescence (ECL plus kit, Perkin Elmer which contains the substrate for horseradish peroxidase, HRP) ^3-5^. Blots were incubated overnight with anti cleaved caspase 3 antibody (caspase 3, rabbit polyclonal, 1:1000 Cell signaling cs9662) an washed and incubated with a secondary antibody HRP-conjugated goat anti-rabbit IgG or a goat anti-mouse IgG for 2 h. As a protein loading control, β-actin was used.

Digital images were obtained with a Syngene G-Box automated system and analyzed by densitometry using the software UN-SCAN-IT™ (Silk Scientific Corporation) ^3-5^

**2.d Evaluation of myocardial apoptosis by the TUNEL assay**^6^

For the identification and quantification of apoptotic cells in the myocardium, fixed cross histological sections of the left ventricle were embedded in 5 μm thick paraffin were stained using the terminal deoxynucleotidyltransferase-mediated dUTP nick end labeling (TUNEL) kit (Roche Co., USA) . The procedure was applied according to the manufacturer's recommendations (incubation time of 60 minutes at 37 ° C for the transferase reaction and pretreating the samples by heating in a microwave oven for 5 minutes with 0.1 M sodium citrate). The cuts were counterstained with hematoxylin. Signal conversion was performed with horseradish peroxidase and diaminobenzidine. Microphotographs were then obtained nder a light microscope at 20X magnification (Eclipse E-800, Nikon) and a digital camera (Coolpix 4500, Nikon) at 0.05 mm2 / field of vision.

For each cut, sixteen consistent fields of vision were analyzed along the circumference of the myocardium. In each field, the number of positively marked nuclei (TUNEL (+)) was counted by differentiating those nuclei located clearly in the center of the cytoplasm of a cardiomyocyte. The total number of visible cardiomyocyte nuclei was also determined to calculate the percentage of TUNEL (+) cardiomyocytes per field of vision. The nuclei were considered as cardiomyocytes whenever they were located in the center of a large cytoplasm cell with characteristic cardiomyocyte morphology in cross section and sharp limits. The procedure was performed by a totally blind observer to the intervention.

**REFERENCES**

[1]. Gabrielli L, Winter JL, Godoy I, McNab P, Padilla I, Cordova S, Rigotti P, Novoa U, Mora I, García L, Ocaranza MP, Jalil JE. Increased Rho-Kinase Activity in Hypertensive Patients With Left Ventricular Hypertrophy. *Am J Hypertens*. 2014 ;27:838-45.

[2]. Ocaranza MP, Gabrielli L, Mora I, Garcia L, McNab P, Godoy I, Braun S, Córdova S, Castro P, Novoa U, Chiong M, Lavandero S, Jalil JE. Markedly increased Rho-kinase activity on circulating leukocytes in patients with chronic heart failure. *Am Heart J* 2011;161:931-37.

[3]. Fierro C, Novoa U, González V, Ocaranza MP, Jalil JE. Simultaneous Rho kinase inhibition in circulating leukocytes and in cardiovascular tissue in rats with high angiotensin converting enzyme levels. *Int J Cardiol*. 2016;215:309-11.

[4]. Rivera P, Ocaranza MP, Lavandero S, Jalil J. Rho kinase activation and gene expression related to vascular remodeling in normotensive rats with high angiotensin I converting enzyme levels. *Hypertension*. 2007;50:792-98.

[5]. Mera C, Godoy I, Ramírez R, Moya J, Ocaranza MP, Jalil JE. Mechanisms of favorable effects of Rho kinase inhibition on myocardial remodeling and systolic function after experimental myocardial infarction in the rat. *Ther Adv Cardiovasc Dis*. 2016;10:4-20.

[6]. Lopez R, Arismendi M, Saez JC, Godoy I, Ocaranza MP. Boldine decreases myocardiac apoptosis post ischemia/reperfusion in rat. *Rev Chil Cardiol* 2011;31:146-154.
